# Evaluation of* Yarrowia lipolytica* Oil for Biodiesel Production: Land Use Oil Yield, Carbon, and Energy Balance

**DOI:** 10.1155/2018/6393749

**Published:** 2018-10-28

**Authors:** Xochitl Niehus, Leticia Casas-Godoy, Francisco J. Rodríguez-Valadez, Georgina Sandoval

**Affiliations:** ^1^Centro de Investigación y Desarrollo Tecnológico en Electroquímica SC. Parque Tecnológico Querétaro Sanfandila, 76703 Pedro Escobedo, Querétaro, Mexico; ^2^Centro de Investigación y Asistencia en Tecnología y Diseño del Estado de Jalisco (CIATEJ), 800 Normalistas Av., Guadalajara 44270, Mexico; ^3^Cátedras CONACYT- Centro de Investigación y Asistencia en Tecnología y Diseño del Estado de Jalisco (CIATEJ), 800 Normalistas Av., Guadalajara 44270, Mexico

## Abstract

Oils from yeasts have emerged as a suitable alternative raw material to produce biodiesel, due to their similar composition to common raw materials such as vegetable oils. Additionally, they have the advantage of not competing with human or animal feed, and, furthermore, they do not compete for arable land. In this work, a carbon and energy balance was evaluated for* Yarrowia lipolytica* as a model yeast, using crude glycerol from biodiesel as the only carbon source, which improves biodiesel overall yield by 6%. The process presented a positive energy balance. Feasibility of yeast oil as biodiesel substrate was also evaluated by determination of the lipid fatty acid profile and cetane number. Moreover, a comparison of oil yields, in terms of land use, between vegetable, microalgae, and yeast oils is also presented. The results showed that* Y. lipolytica* oil yield is considerably higher than vegetable oils (767 times) and microalgae (36 times).

## 1. Introduction

Biodiesel has become the most sustainable and renewable alternative to fossil diesel. It is defined as a mixture of free fatty acid alkyl esters, usually obtained from vegetable oils and animal fats [1]. The use of these oils as raw materials accounts for around 88% of the production costs [2] and has also generated polemics about the usage of edible oils to produce biofuels while there is still hunger in the world. Therefore, many studies are focused on the utilization of lower-cost and nonedible feedstocks, such as waste or nonedible oils [1]. In this regard, microbial oils have emerged as alternative raw materials. Microbial oils are defined as the oils produced by oleaginous microorganisms, i.e., microorganisms able to accumulate more than 20% of their dry cell weight (DCW) as lipids in the form of droplets inside the cells [3]. This accumulation is mainly due to an excess of carbon (C) source and a limiting amount of another nutrient, such as nitrogen (N) [4, 5]. Lipids from yeasts are mainly triacylglycerols, which can be compared, in terms of their chemical composition, to lipids obtained from plant oilseeds (vegetable oils). Furthermore, yeasts can use a wide range of nutrient sources, including industrial wastes, which could reduce production costs. The main coproduct of biodiesel is crude glycerol, which before a highly cost purification process has a limited amount of applications. The increased availability of crude glycerol, resulting from the growing production of biodiesel, has attracted the attention of researchers. Several studies are focused on adding value to this coproduct by using it as a substrate for microbial cultures in biotechnological processes [6–10].

Among the most studied oleaginous yeasts, we can find* Yarrowia lipolytica*, a dimorphic yeast with a known genome [11]. In this work a recently isolated* Y. lipolytica* strain was cultured in crude glycerol as a model to evaluate its biomass energy potential and carbon balance. In addition, fatty acid profile and cetane number were evaluated. Finally, a comparison of land use oil yields between vegetable, microalgae, and yeast oils is presented. To our knowledge, this is the first report of this kind that includes yeast oils.

## 2. Materials and Methods

### 2.1. Yeast Strain

The yeast strain used for the study was a* Yarrowia lipolytica*, which was previously isolated in our lab and selected by its lipid content and versatility to grow in different substrates. Yeast identification was performed by PCR-RFLP analysis according to Segura et al. [12]. This wild type yeast was deposited under the Budapest Treaty in the Agricultural Research Service Culture Collection (NRRL) with the number NRRL Y-50997.

### 2.2. Production of Yeast Oils

Yeast oils were produced in 500 mL Erlenmeyer flasks containing 100 mL of nitrogen limited medium. A nitrogen limited medium was prepared according to Suutari et al. [13], using crude glycerol instead of glucose. Cultures were inoculated with a 10 mL overnight preculture grown in YPD (20 g/L glucose, 20 g/L peptone, 10 g/L yeast extract). Liquid cultures were performed in duplicate on orbital shakers at 250 rpm and 30°C, for 72 h. Samples were taken during the production and frozen until further analysis. Crude glycerol was obtained from a local biodiesel producer. Unless otherwise stated, commercial grade chemicals were purchased from Sigma-Aldrich (Mexico).

### 2.3. Analytical Methods

During yeast oil production cell growth, lipid content, nitrogen, and glycerol consumption were analyzed for all the samples taken. Cell growth or biomass was determined by measuring the DCW after drying to constant weight. The lipid content was measured by the extraction of lipids from the cells using the method proposed by Schneiter et al. [14]. Lipid percentage was calculated using DCW and lipid content. Nitrogen consumption was reported as the sum of inorganic and organic nitrogen present in the supernatant, which were measured by the colorimetric techniques proposed by Chaney et al. [15] and Sun et al. [16], respectively.

Biomass elemental composition was measured in washed and dried samples obtained from the final yeast culture and was determined using an EA 1108 Fisons instrument model EA1108 CHNS. Fatty acid profile of the final culture was analyzed in a gas chromatograph equipped with flame ionization detector (Perkin Elmer® model AutoSystem XL, USA) using an Alltech AT-WAX 30mm × 0.25mm × 0.25 *μ*m capillary column (J and W Scientific, USA) and nitrogen as the carrier gas. Fatty acid methyl esters were identified by comparison of their retention times with commercial standards, and the lipid profile was obtained using methyl heptadecanoate as internal standard. All the measurements were performed in duplicate.

### 2.4. Mass and Energy Balance Equations

A mass balance in a batch or flask microbial culture can be described by the following equation [17]:

(1) $$\begin{array}{c} \frac{dX}{dt}=\mu X \end{array}$$

where X is the biomass concentration (g/L), t is time (h), and *μ* is the specific growth rate (h^−1^). During the exponential growth phase, *μ* is considered to be constant. Integrating (1) with the initial condition X = X_0_ at t = t_0_, it becomes [17]

(2) $$\begin{array}{c} \mu t=\ln\frac{X}{X_{0}} \end{array}$$

Using (2), *μ* can be easily calculated.

In order to perform an energy balance of the microbial oil produced, a similar procedure to that proposed by Anschau et al. [18] was used and is described as follows.

Elemental balance equation of microbial growth can be written as [17]

(3) CwHxOyNz+aO2+bHgOhNi→cCHαOβNδ+dCO2+eH2O+fCjHkOlNm

where the terms C_*w*_H_*x*_O_*y*_N_*z*_, H_*g*_O_*h*_N_*i*_, CH_*α*_O_*β*_N_*δ*_, and C_*j*_H_*k*_O_*l*_N_*m*_ correspond to substrate elemental composition, nitrogen source, cell biomass, and extracellular product, respectively. Since microbial oils are mainly intracellular (accounted in the cell biomass term), the product term can be neglected. The carbon balance can be expressed as

(4) $$\begin{array}{c} 1=c+d \end{array}$$

where* c* is the carbon biomass yield and* d* is related to respiration ratio. Moreover, degrees of reduction represent the electrons per unit of carbon in the substrate and biomass, respectively, relative to the valence state of the carbon in each molecule. These values correspond to 4 for carbon atoms, 1 for hydrogen atoms, -2 for oxygen atoms, and -3 for nitrogen atoms [17]. In this sense, electron availability balance of equation (3) is given by

(5) $$\begin{array}{c} \gamma_{S}+a\left(\;-4\;\right)=c\gamma_{B} \end{array}$$

where

(6) γS=4+xw−2yw

(7) γB=4+α−2β−3δ

Subscripts identify the substrate (S) and biomass (B), respectively. A high degree of reduction denotes a low degree of oxidation which relates the relative electrons gained (reduction) when the substrate carbon becomes biomass.

On the other side, dried biomass and glycerol elemental composition were used to calculate the term* η*, which represents biomass formation energy yield and corresponds to the ratio of biomass heat of combustion to the heat of combustion of the corresponding amount of substrate metabolized [19]. According to Erickson et al. [20] *η* can be related with the biomass to substrate yield (Y_X/S_) as follows:

(8) $$\begin{array}{c} \eta =\left(\;\frac{\sigma_{B}\gamma_{B}}{\sigma_{S}\gamma_{S}}\;\right)Y_{X/S} \end{array}$$

where *σ*_B_ and *σ*_S_ are carbon weight fractions in biomass and substrate, respectively.

In order to complete biomass energy potential, according to Meier et al. [21], combustion heat (*Q*_c_) can be calculated from biomass elemental composition using the following equation with a standard error of 5%.

(9) $$\begin{array}{c} Q_{c}=33.5\left(\;C_{F}\;\right)+142.3\left(\;H_{F}\;\right)-15.4\left(\;O_{F}\;\right)-14.5\left(\;N_{F}\;\right) \end{array}$$

in which C_F_, H_F_, O_F_, and N_F_ are weight fractions of each element in the biomass.

### 2.5. Cetane Number Calculation

The cetane number (CN) was calculated empirically using a multiple regression equation [22].

(10) $$\begin{array}{c} CN=1.068\Sigma \left(\;CN_{i}\times W_{i}\;\right)-6.747 \end{array}$$

where CN represents the cetane number of the final mixture, W_i_ is the mass fraction of individual FAME, and CN_i_ is the CN of the pure FAME.

(11) CNiunsaturated  fatty  acids=109−9.292X+0.354X2

(12) CNisaturated  fatty  acids=−107.71+31.126X−2.042X2+0.0499X3

where X is the carbon number of each individual fatty acid.

## 3. Results and Discussion

### 3.1. Culture Kinetics and Carbon Balance

**Figure 1:**
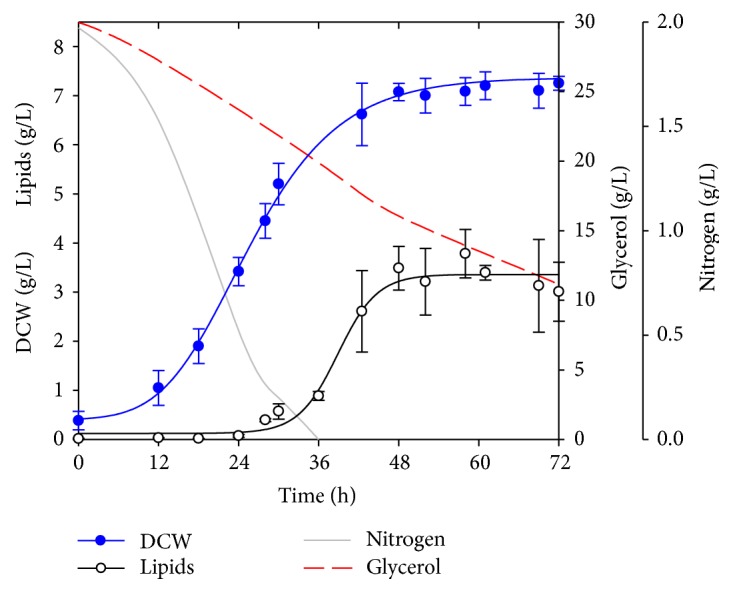
*Y. lipolytica* flask culture kinetics.

The yeast* Y. lipolytica* was cultured for 72 h in the nitrogen limited medium.  Figure 1 presents the kinetics of biomass and lipid production, as well as glycerol and nitrogen consumption. It can be observed that nitrogen was completely consumed at 36 h, while only 63% of the initial glycerol was consumed at the end. This indicates that the yeast might be able to continue consuming glycerol, but since biomass reached the stationary growth phase at 48 h, the cultures were stopped at 72 h. Lipid production in the yeast increased almost at the same time that nitrogen was completely consumed, which is in accordance with previous reports [4] and continued increasing until 48 h where lipid production reached a stationary phase. Changes in pH were monitored every 12 h (data not shown), pH diminished only from 5.5 to 5 after 72 h.  Table 1 shows the maximum biomass, lipid percentage, lipid content, and lipid productivity achieved. Lipid percentage obtained is higher than other reports under similar conditions using glycerol as carbon source [6–10].

In terms of glycerol consumption, the maximum theoretical lipid to substrate yield (Y_L/S_) value is 0.3 g lipids/g glycerol; this is based on carbon balance since 32 moles of glycerol are used to produce 1 mole of triglyceride [23]. The value obtained in Table 1 corresponds to 63% of the theoretical value and implies that 63% of the glycerol used is directly converted to lipids. This is a promising result, considering that the process can still be optimized. Also, with this result, the overall biodiesel yield can be improved by 6%, since residual carbon atoms, in the form of glycerol, can be returned to the same process.

It is worth mentioning that this wild type strain of* Y. lipolytica* is among the best when cultured in crude glycerol in terms of lipid content and lipid percentage [24, 25] but outstands in lipid productivity. Furthermore, culture conditions and medium composition can be optimized in order to improve final yields.

### 3.2. Energy Balance

Biomass elemental composition, at the end of the culture, was 50.2% carbon, 7.63% hydrogen, 34.16% oxygen, and 5.96% nitrogen, which corresponds to an elemental formula of CH_1.82_O_0.51_N_0.1_. For other species of oleaginous yeast strains the elemental composition has been reported as relatively constant (≈ 75.6% carbon, 11.6% hydrogen, and 12.7% oxygen) [26] but this can be applied only for a specific carbon source (ethanol) in similar culture conditions.

Using this analysis, the data showed in Table 1 and (1) to (9), the following parameters are obtained (Table 2).

The value obtained for the biomass formation energy yield (*η*) indicates that 44% of the energy supplied by the glycerol is converted to biomass and microbial lipids. The combustion heat obtained (*Q*_c_) corresponds to approximately 47% of the energy content in fossil diesel (45.4 kJ/g [18]), indicating the potential of this oleaginous yeast for biodiesel production. This is in agreement with a previous report [26] in which, for a yeast with a lipid content of 64%, the energy value of dry biomass reached 73% of biodiesel oil.

Moreover, we calculate that the energy cost of our process is close to 5 kJ/g, and considering that we obtain 21.5 kJ/g, process overall energy balance is positive and corresponds to 4.5 times more energy obtained. This value is similar to the energy obtained in a biodiesel production process using palm oils (the vegetable oil with the highest yield), which corresponds to 4.7 times [27] the energy employed, but it is worth mentioning that the land used is higher. Even tough crude glycerol can be used to produce other biofuels, Zhang et al. [28] found that the use of crude glycerol for biodiesel production is the only process that has an energy gain, with a positive energy balance and conversion efficiency greater than 1, which is in agreement with our results.

### 3.3. Fatty Acid Profile

Lipid fatty acid profile allows determining possible applications. Specifically, for biodiesel, fatty acid profile has a direct impact in the final product properties such as cetane number, viscosity, density, and fusion temperature. In general, a higher saturated fatty acid content corresponds to higher viscosity, density, and fusion temperature. Therefore, it is desirable that raw materials for biodiesel production are rich in monounsaturated fatty acids such as oleic acid.* Y. lipolytica* oil's fatty acid profile is presented in Table 3. Since the obtained oil is rich in oleic acid, this constitutes a good biodiesel raw material.

From the fatty acid profile obtained in Table 3, cetane number was calculated according to (10) to (12). The value calculated for this* Y. lipolytica *oil was 61.5 which complies with the ASTM D976 (CN≥48). This result confirms that the obtained oil is a suitable alternative raw material to produce biodiesel.

### 3.4. Comparison of Oil Yields in Terms of Land Use

**Figure 2:**
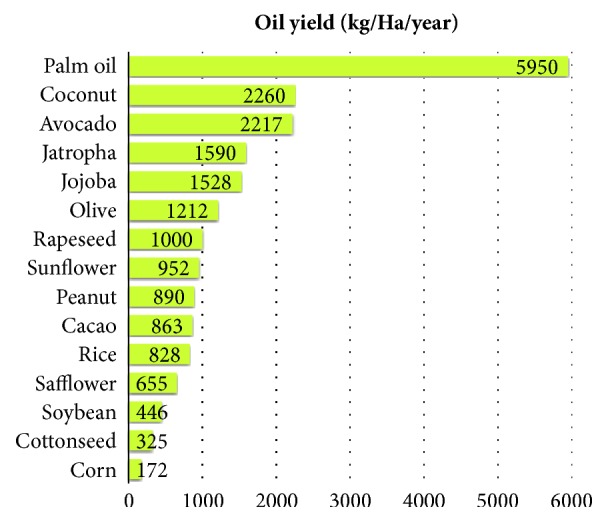
Annual oil yields of different vegetable crops. Adapted from Tickell et al. [33] and Ma et al. [31].

Main raw materials to produce biodiesel are lipids or oils rich in triglycerides. These are usually obtained from edible crops that represent an ethical debate about the use of food, water, and arable land for biofuels while there is still hunger in the world. Recently nonedible oils from crops that do not require high amounts of water neither very fertile land, such as jatropha [29], have been proposed as alternative raw materials. Nevertheless, as shown in Figure 2, when taking into account oil yields in terms of land use, palm oil is the best crop. However, its plantation in places such as Borneo has resulted in a deforestation problem, and many countries have adopted a no palm oil products policy until it is produced in a sustainable way.

**Figure 3:**
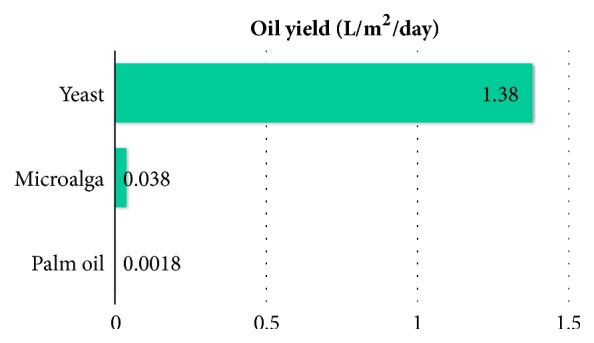
Oil yields per unit of area (m^2^) per day.

In this sense, microbial oils have attracted attention as alternative biodiesel raw materials. Among microbial oils many studies have been performed in microalgae, and it has been reported that they present higher land use yields than palm oil [30], even though it must be considered that most of them require a continuous light source in order to promote photosynthesis, which makes the process less energy effective during the night. Also, lipid concentration in microalgae is in the order of milligrams per liter while in oleaginous yeasts, such as the* Y. lipolytica* used in this work, it is in the order of grams per liter and they can be cultured in stirred tank bioreactors without light. Regarding all the above and using the data obtained in this work, oil yields per unit of area per day were calculated for palm [31], microalga [30], and yeast oils. The results are presented in Figure 3. As can be seen, although microalga yield is more than 20 times higher than palm oil, the oil yield in the yeast is 36 times higher than microalga and 767 times higher than palm oil. In this way yeast oils have great potential as alternative raw material to produce biodiesel. It is worth mentioning that oil yields in* Y. lipolytica* can be improved by culture optimization and genetic modifications [11, 32], so the yields can be even higher.

## 4. Conclusions

A positive energy balance is obtained when producing microbial oils with* Y. lipolytica* and crude glycerol as the only carbon source. Furthermore, lipids produced are suitable as alternative raw material for biodiesel production and the overall process yield is improved by 6%, when returning crude glycerol in the same process. Additionally, in terms of land use, the oil yield obtained is considerably higher than vegetable oils and microalgae. In conclusion, yeast oils have great potential to be used as alternative biodiesel raw material. Further studies about process optimization, economics, and environmental impacts are currently under study.

## Figures and Tables

**Table 1:** Maximum biomass, lipid percentage, and lipids in flasks cultures.

**Parameter**	**Value**
Biomass (DCW, g/L)	7.2±0.2
Lipid percentage (% DCW)	53±0.7
Lipids (g/L)	3.8±0.3
Y_L/S_ (g lipids/g glycerol)	0.19±0.01
Y_X/S_ (g biomass /g glycerol)	0.36±0.01
*μ* (h^−1^)	0.06±0.002
Productivity (g lipids / L*∗*h)	0.08±0.005

Y_L/S_: lipid to substrate yield; Y_X/S_: biomass to substrate yield; *μ*: specific growth rate.

**Table 2:** Calculated parameters for energy balance.

**Parameter**	**Value**
*γ*_S_	4.67
*γ*_B_	4.5
*η*	0.44
*Q*_c_ (kJ/g)	21.5

*γ*_S_ = substrate (glycerol) degree of reduction, *γ*_B_ = biomass degree of reduction, *η* = biomass formation energy yield, and *Q*_c_ = combustion heat.

**Table 3:** Oil fatty acid profile.

**Fatty acid**	**Content** **(**%** weight of total lipid)**
Palmitic (C16:0)	14.9
Stearic (C18:0)	11.1
Oleic (C18:1)	55.1
Linoleic (C18:2)	18.5
Linolenic (C18:3)	0.3

## Data Availability

The data used to support the findings of this study are included within the article.
